# Emotional Prosody Processing in Schizophrenic Patients: A Selective Review and Meta-Analysis

**DOI:** 10.3390/jcm7100363

**Published:** 2018-10-17

**Authors:** Yi Lin, Hongwei Ding, Yang Zhang

**Affiliations:** 1 Institute of Cross-Linguistic Processing and Cognition, School of Foreign Languages, Shanghai Jiao Tong University, Shanghai 200240, China; carol.y.lin@sjtu.edu.cn; 2 Department of Speech-Language-Hearing Sciences & Center for Neurobehavioral Development, University of Minnesota, Twin Cities, MN 55455, USA

**Keywords:** emotional prosody processing, schizophrenia, meta-analysis

## Abstract

Emotional prosody (EP) has been increasingly recognized as an important area of schizophrenic patients’ dysfunctions in their language use and social communication. The present review aims to provide an updated synopsis on emotional prosody processing (EPP) in schizophrenic disorders, with a specific focus on performance characteristics, the influential factors and underlying neural mechanisms. A literature search up to 2018 was conducted with online databases, and final selections were limited to empirical studies which investigated the prosodic processing of at least one of the six basic emotions in patients with a clear diagnosis of schizophrenia without co-morbid diseases. A narrative synthesis was performed, covering the range of research topics, task paradigms, stimulus presentation, study populations and statistical power with a quantitative meta-analytic approach in Comprehensive Meta-Analysis Version 2.0. Study outcomes indicated that schizophrenic patients’ EPP deficits were consistently observed across studies (*d* = −0.92, 95% CI = −1.06 < δ < −0.78), with identification tasks (*d* = −0.95, 95% CI = −1.11 < δ < −0.80) being more difficult to process than discrimination tasks (*d* = −0.74, 95% CI = −1.03 < δ < −0.44) and emotional stimuli being more difficult than neutral stimuli. Patients’ performance was influenced by both participant- and experiment-related factors. Their social cognitive deficits in EP could be further explained by right-lateralized impairments and abnormalities in primary auditory cortex, medial prefrontal cortex and auditory-insula connectivity. The data pointed to impaired pre-attentive and attentive processes, both of which played important roles in the abnormal EPP in the schizophrenic population. The current selective review and meta-analysis support the clinical advocacy of including EP in early diagnosis and rehabilitation in the general framework of social cognition and neurocognition deficits in schizophrenic disorders. Future cross-sectional and longitudinal studies are further suggested to investigate schizophrenic patients’ perception and production of EP in different languages and cultures, modality forms and neuro-cognitive domains.

## 1. Introduction

Schizophrenia is a severe chronic psychiatric illness affecting people’s educational, vocational and daily performance [1]. More than 21 million people worldwide suffer from this mental disorder, with males (12 million) more common than females (9 million). People with schizophrenia often bear profound dysfunctions in social cognition, which refers to how they think, feel and behave, including the sense of self [2,3]. They are also characterized by distortions in emotions and language [4], which usually result in an isolation in social interactions [5].

Social cognition research has four characteristic features, respectively focusing on mentalism, attempting to understand the causal mechanism of the cognition process, adopting approaches and ideas from multiple disciplines, and concerning with real-world applications [6]. Recent years have witnessed increasing attention on the study of social cognition deficits in clinical populations, including patients with schizophrenia. These studies largely fall into three thematic categories: (1) Theory of Mind, which investigates how participants represent their mental states and make inferences about others’ intentions, (2) emotion perception, which addresses how participants identify and discriminate facial and vocal affect as well as other social signals such as gesture, and (3) attributional style, which is concerned with how participants give explanations to the positive and negative events happening in their lives [6,7]. A line of studies has investigated how social cognition abnormality is related to other domains in schizophrenic patients, such as neurobiology [7], neurocognition [8], functional outcomes [8,9] and negative symptoms [6]. There is a growing amount of evidence showing schizophrenic patients’ impairments in social cognition, which can be regarded as a powerful predictor of patients’ impaired social functioning [7,10]. Some researchers further identified comparable social cognition disorders between schizophrenic patients and patients with other psychotic disorders such as bipolar disorder [11], and highlighted the importance of continuous psychosocial support and social cognition analysis techniques and training programs [12,13,14].

Successful interpersonal communication requires an individual to be able to interpret and respond to pertinent social signals of others in dynamic contexts. As a key domain of social cognition and an organic integration of emotions and language, emotional prosody (EP), also known as affective prosody, is an important component of communication conveying speakers’ feelings and intent through variations of parameters such as pitch, stress and duration [15]. Emotional prosody processing (EPP) refers to the perception and production of EP, the study of which can contribute to the clinical diagnosis and treatment as well as in-depth research on participants’ processing of both linguistic and non-linguistic information in communication. The EPP performance of patients with psychotic disorders have been a focal point in recent years’ research, involving people suffering from schizophrenia, borderline personality disorder, autism spectrum disorder, bipolar disorder, depression, and schizoaffective disorder [11,16,17,18,19]. In these studies, affect perception ability was shown to have an association with social functioning and play an increasingly important role in interpersonal relations.

To date, a number of studies have discovered that people with schizophrenia show reduced emotional expressivity and impaired emotional prosodic comprehension [20,21,22]. Although a complete inability of EPP in terms of the perceptual, cognitive and social functions is unlikely in these individuals, the observed differences in both areas of EP comprehension and production deserve systematic exploration to determine their functional and clinical significance, which may lead to the development of novel and alternative ways to approach social cognition deficits in schizophrenic patients and patients with psychiatric disorders in general. Considering that emotion is a significant component of social cognition, schizophrenic patients’ deficits at this level may result in more interpersonal misunderstanding or inappropriate social behaviors [23], thus preventing them from assimilating into a larger social community.

One challenging problem that faces the field of schizophrenia research and clinical practice is that although cognitive impairment generally co-occurs with psychosis, it may reflect processes or an epiphenomenon independent of genetic and biological factors that lead to the mental disorder itself. In terms of EPP, it remains unclear whether there is sufficient and strong evidence to determine EPP impairment as a pervasive and intrinsic defining feature of the psychopathology of schizophrenia. To our knowledge, few studies have systematically examined schizophrenic patients’ EPP except a meta-analysis that only focused on behavioral studies undertaken between 1980 and 2007. The scope and depth of that review are limited as it did not take into account recent advancements using new experimental techniques and did not evaluate the influence of different task paradigms [24]. With the advancement of neuroimaging techniques, it is of great necessity to integrate or reconcile findings from the latest brain research studies with behavioral studies under different experimental designs in order to gain a better understanding of schizophrenic patients’ EPP deficits, including heterogeneity in performance and the underlying neural markers. Presumably, in the case of EPP, various dissociable component behavioral elements can be examined, including input-related attentional and cognitive processes and output-related motor control processes, to identify potential specific target components for therapeutic interventions. However, there is still a lack of a comprehensive overview to examine the additive and integrative nature of these components in this regard. In particular, a synopsis of studies is in need to summarize (a) the central topics, research design and statistical analysis of the latest related studies, (b) schizophrenic patients’ EPP performance under different experimental conditions influenced by variations in task paradigms, response options, stimulus modalities, participants’ demographic, clinical and cognitive backgrounds, and (c) the physiological and neuroanatomical basis in terms of hemispheric, spatial and temporal processing of EPP in patients with schizophrenia.

As methods of cognitive and functional assessment are fundamental to interpretations of the research findings on schizophrenia, it is necessary to periodically reflect upon the new trends of developments and existing tools and protocols that may need modifications to further advance the field. By analyzing selected behavioral and neuroscientific studies via five major online databases, our current selective review aims to (a) summarize the characteristics of the latest studies, (b) determine the magnitude of impairments in EPP of schizophrenic patients by calculating the effect size in terms of different task paradigms, and (c) identify the potential factors and neural mechanisms influencing schizophrenic patients’ EPP performance. We hope to deepen the understanding of the nature of EPP impairments in schizophrenic patients as a core deficit and contribute to the diagnosis and rehabilitation of this psychiatric disorder. This review also serves to provide suggestions and implications for future research on this promising topic for experts from multidisciplinary areas, including speech and language, psychotic disorder, psychosocial mediation, and neuroimaging.

## 2. Method

### 2.1. Inclusion and Exclusion Criteria

#### 2.1.1. Types of Studies

The studies eligible for inclusion in this review had to investigate EPP of patients with schizophrenia in comparison with a control group(s). The following types of studies were included:

(a) cross-sectional studies comparing schizophrenic patients versus healthy controls or/and versus a clinical comparison group (i.e., patients with a different psychiatric disorder or neurological condition);
(b) longitudinal studies comparing participants’ performance before versus after schizophrenia.

We only included studies containing at least one type of the six basic emotions classified by Ekman [25]: anger, disgust, fear, happiness, sadness and surprise. Complex or social emotions (such as contempt, sarcasm, trustworthiness, and embarrassment) were therefore not taken into consideration in this review. The studies needed to use behavioral tasks such as identification, discrimination, labeling and rating. In these tasks, participants were required to evaluate the stimuli based on the emotional information conveyed by the stimuli. The stimuli could be of any form (e.g., words, sentences, pictures, sounds) and of any modality (e.g., auditory, visual). Studies exclusively focusing on mood, emotional memory, emotional production and facial emotional processing were excluded. Finally, reviews, editorials, conceptual papers and book chapters with no original data were excluded, so were the abstracts (primarily conference papers) with no access to full texts.

#### 2.1.2. Types of Participants

Given that schizophrenia typically begins in early adulthood or late adolescence, studies that involved adult patient populations (over 18 years old) were eligible for this review. Participants must have a clear diagnosis of schizophrenia through Diagnostic and Statistical Manual of Mental Disorders (DSM) or WHO International Classification of Disorders (ICD). We also excluded studies involving participants with co-morbid physical or psychological diseases, uncorrected auditory or visual impairments or any neurocognitive disorders. Studies concerning schizoaffective disorders were included whereas schizotypal personality disorders (STPD) were not taken for review since the classification of STPD has not been unified in DSM-V and ICD-10 [26]. The current review also excluded prodromal schizophrenia patients so that we can provide a more accurate description of people suffering from schizophrenia at the time of the experiments. Additionally, demographic information such as gender, education, the duration and severity of illness evaluated by PANSS (Positive and Negative Syndrome Scale) should be specified as detailed as possible [27]. The ramifications of our inclusion and exclusion criteria for this selective review are further elaborated in the discussion section.

#### 2.1.3. Types of Measures

Studies must adopt an experimental or a quasi-experimental method and report a quantitative measure of EPP.

### 2.2. Searching Strategies for Identification of Studies

Comprehensive research was conducted electronically through the following databases: Web of Science Core Collection, MEDLINE, PsycINFO, PsycARTICLES and Psychology and Behavioral Sciences Collection. The first two databases were retrieved from Web of Science (WOS) while the rest were accessed using EBSCO Academic Source Complete. The advanced search keywords were “emotional prosody processing” OR “affective prosody processing” AND “schizophrenia”. The search included studies with publication dates up to April 2018. For each study included in the review, we also conducted manual searches of reference lists so as to identify additional potential studies.

### 2.3. Data Collection and Analysis

#### 2.3.1. Selection of Studies

**Figure 1:**
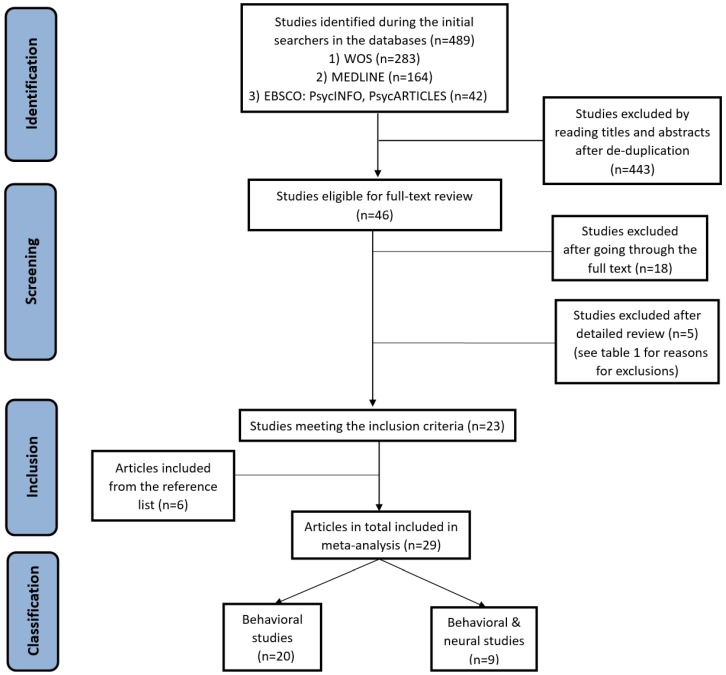
Flowchart of selecting studies for review.

Initial searches in the databases returned a total of 489 studies. The title and abstract of each study were first checked in accordance with the inclusion and exclusion criteria, which resulted in 46 candidates after removing duplicated citations across the search results. The full text of these 46 papers was then examined, and another 5 studies were excluded after detailed checking (see Table 1 for reasons for exclusion). The screening process discovered 23 studies that fully met our selection criteria. After reading the full contents in detail, six additional studies were identified from the reference lists in the 23 articles. Thus, the total number of the selected publications for the final meta-analysis is 29. The flowchart of study selection is presented in Figure 1.

#### 2.3.2. Data Extraction

Data were extracted concerning the following key elements: (a) general characteristics of the studies, (b) statistical analysis and (c) study outcome. We designed a data collection form to record a range of aspects, including study type (behavioral or neurological), sample size, stimulus presentation, task paradigm, response option, demographic information, methods of analysis, reporting of statistical results, and major findings.

#### 2.3.3. Data Synthesis

A narrative synthesis method was employed to describe and compare the selected studies by summarizing their research topics, task paradigms, stimulus presentation, response options, patients’ backgrounds, statistical analysis methods, participants’ overall and single emotion processing performance. A quantitative meta-analytic approach was adopted to calculate the effect sizes (Cohen’s d) for the difference in EPP between schizophrenic patients and healthy controls based on the reported statistics. Cohen’s d was calculated with an online meta-analysis effect size calculator developed by David Wilson [58]. Means and standard deviations, if reported in the selected research, were used for calculation of individual studies. Otherwise, reported t or F statistics were used. The values of effect size were calculated in terms of two experimental paradigms: identification and discrimination test. For the studies that applied more than one task measuring EPP of patients and healthy controls or more than one subtype of schizophrenic patients in each paradigm [31,33], the performance results in these tasks or patient groups were averaged and taken together as one value.

The computed effect sizes of 29 individual studies were then further analyzed using Comprehensive Meta-Analysis Version 2.0 [59]. Studies were weighted in order to control the differences in sample size. The mean effect sizes across all studies (including both identification and discrimination tasks) were calculated and presented in a forest plot using the random-effects model since the selected studies differed from one another in study populations which could influence the treatment effect [60]. We categorized the cutoff points of effect size as small (*d* = 0.2), medium (*d* = 0.5) or large (*d* = 0.8) [61]. Publication bias was also assessed graphically using a funnel plot.

## 3. Results

### 3.1. General Characteristics of the Included Studies

Table 1 summarizes the general characteristics of the related studies, including study types, topics, task paradigms, stimulus modalities and forms, response options, study participants, patients’ demographic and clinical information, statistical analysis methods and reasons for exclusion from meta-analysis.

#### 3.1.1. Study Topics

Out of the 29 studies selected for review, all employed behavioral tasks, and only 9 included neurophysiological data. While all the 29 studies examined the perception of emotional prosody in schizophrenic patients, only 2 involved the production of EP [28,31]. Therefore, we will mainly discuss the emotional prosody perception in this review.

One central topic of the behavioral studies was the relationship between EPP and its influential factors. Non-linguistic influential factors of EPP such as emotion clarity, gender, IQ, types and models of processing, psychotic symptoms, hemispheric dysfunctions and neuropsychological function had been focal points of attention [28,34,35,36,38,40,46,54,57]. In recent years, a few studies also examined linguistic factors influencing patients’ performance such as tone of voice [43] and semantic contents [47]. Another main topic was the relationship between emotional prosody and other cognitive and social indices such as social functioning [29], auditory processing [30,41] and auditory hallucinations [31,33,44].

Among the selected cognitive neuroscience studies, magnetic resonance imaging (MRI) and functional magnetic resonance imaging (fMRI) were employed to study the hemispheric processing and spatial localization of EPP [32,37,53,55]. Event-related potential (ERP) correlates were also under the exploration of several studies to examine the temporal processing during emotional prosody perception [42,45,50,51,53].

#### 3.1.2. Task Paradigms, Stimulus Presentation and Response Options

The test paradigms of EPP in the selected studies generally fell into two major categories: identification task (24 studies) and discrimination task (9 studies) with some studies using both tasks. In the identification tasks (also called explicit EPP task), participants were usually instructed to recognize the emotion that the voice expressed in a semantically neutral sentence [62]. To measure EPP on a more implicit level, discrimination tasks were often used, in which participants were asked to differentiate either the stimuli presented in pairs (5 studies) or judge congruency between spoken verbal material with either congruent or incongruent emotional valences (4 studies). The former tasks were usually combined with a dichotic listening paradigm where participants were asked to attentively focus on one of the stimuli when they were presented simultaneously to both ears. The latter case was analogous to Stroop tests, and the participants were instructed to focus on either prosodic congruency or semantic congruency in separate test trials.

Verbal stimuli with semantic content (SSC) and stimuli that kept the emotional prosody information without semantic content (SPP) were employed in the selected studies. SSC varied in different linguistic units, covering syllables, words, phrases and sentences. SPP were also presented in various forms such as vocal sounds, frequency-modulated (FM) tones, asyllabic sounds, non-words and meaningless sentences. All studies adopted auditory stimuli and a few also tested facial emotion recognition in their studies. However, only one study combined visual stimuli (pictures) and auditory stimuli (sounds) simultaneously in the same task [50].

#### 3.1.3. Study Participants

The studies were conducted in a number of countries, including the United States (13), Australia (3), France (3), Japan (2), China (2), Switzerland (2), Belgium (1), The Netherlands (1), Austria (1), and Poland (1). Ethnicities of participants in each country were not explicitly indicated in all studies. English (16) was the major language used in the selected studies, followed by French (2), German (2), Japanese (2), Mandarin Chinese (2), Polish (1), and Dutch (1), whereas one study did not specify the task language. Interestingly, studies in non-tonal languages (e.g., English, German, Dutch, Japanese and Polish) significantly outnumbered tonal languages (e.g., Mandarin Chinese), and many more studies were conducted in stress-timed languages (e.g., English, German, Polish and Dutch) than syllable-timed languages (e.g., French and Mandarin Chinese). All studies chose healthy participants as control. There was one study which not only used healthy controls, but also selected patients with left brain damage (LBD) and right brain damage (RBD) for comparison of patient profiles in affective-prosodic deficits [28]. In two other studies, patients with depression and bipolar disorder were also included as a comparison group in addition to healthy controls [11,36].

As described in the selected articles, different types of schizophrenia or schizoaffective disorders were involved, including chronic schizophrenia (CS), paranoid schizophrenia (PS), schizophrenia with auditory verbal hallucination (SAVH), a disorganized type, and a residual subtype. There were five subtypes of schizophrenia in the fourth edition of the Diagnostic and Statistical Manual of Mental Disorders (DSM-IV), respectively known as paranoid, disorganized, catatonic, undifferentiated, and residual. For instance, disorganized schizophrenia is characterized by disorganized symptoms including speech, behavior, and flat or inappropriate affect, whereas residual schizophrenia refers to much alleviated cases in which the patient no longer shows the prominent schizophrenic symptoms but some hallucinations or idiosyncratic behaviors may still be present. One important note here is that the selected studies in our review cover the period of 2001 to 2018. Within this time frame, there have been drastic changes from the fourth edition to the current fifth edition (DSM-V). In DSM-V, however, the five subtypes of schizophrenia are no longer included as the American Psychiatric Association determined that these distinctions were not helpful to clinicians because patients’ symptoms may overlap and change from one subtype to another.

#### 3.1.4. Reporting of Key Demographic and Clinical Information of Schizophrenic Patients

Table 1 also illustrates the reporting of some basic information of the schizophrenic patients. The data presented this table involve patients who participated in the corresponding studies regardless of different tasks paradigms. The sample sizes vary tremendously with the number of schizophrenic patients ranging from 15 to 111 with the mean standing at 39.3 and standard deviation at 24.3, and the average percentage of male was 64.0%. Age was reported by all studies and more than half of the studies included information such as mean years of education and IQ. Handedness, participants’ socioeconomic status, parents’ socioeconomic status, race, parental education, musical background and employment were additional information only recorded by several studies [41,51,53].

The included studies reported duration of illness (20) twice more frequently than age of onset (8). The Positive and Negative Syndrome Scale (PANSS) (18), the Scale for the Assessment of Positive Symptoms (SAPS) (8), the Scale for the Assessment of Negative Symptoms (SANS) (11) and the Brief Psychiatric Rating Scale (BPRS) (5) were widely-acknowledged as systematic protocols for studies to retrieve symptomatic data, but none of them were administered by more than 60% of the selected studies. Twenty-three studies contained medical information with either the type or dose of medication specified.

#### 3.1.5. Statistical Analysis Methods and Reporting of the Results

A variety of combined statistical methods for data analysis were adopted in the 29 selected studies for the current review. Parametric tests were typically used for between and within group statistical comparisons, including Analysis of Variance (ANOVA), Multivariate Analysis of Variance (MANOVA), Analysis of Covariance (ANCOVA), Independent-samples *T* test, Paired-samples *T* test, and One-sample *T* test. To further probe the interaction effects, post hoc tests were conducted, including Tukey test and Student–Newman–Keuls test (SNK), and Bonferroni correction was generally adopted for multiple comparisons. Non-parametric tests were also administered for categorical variables in some studies, which included Chi-square test, Mann–Whitney U test (M-U) and Wilcoxon Rank test.

Approximately half of the studies (15 out of 29) calculated Pearson’s and Spearman’s rank-order correlation coefficients to examine the correlation between variables. Other studies also used more sophisticated approaches to address the relationships between multivariate measures, including multiple linear regression, multivariate linear regression and principal component analysis (PCA). In analyzing neurophysiological data [37,53,55], Statistical Parametric Mapping (SPM) appeared to be most frequently applied. Depending on the research hypotheses of the studies, laterality measures and voxel wise correlation approach were also used [32,37]. In general, *p* < 0.05 was adopted for reporting statistical significance, and other significance levels with smaller alpha values such as 0.01 were also used.

### 3.2. Performance of Emotional Prosody Processing

**Figure 2:**
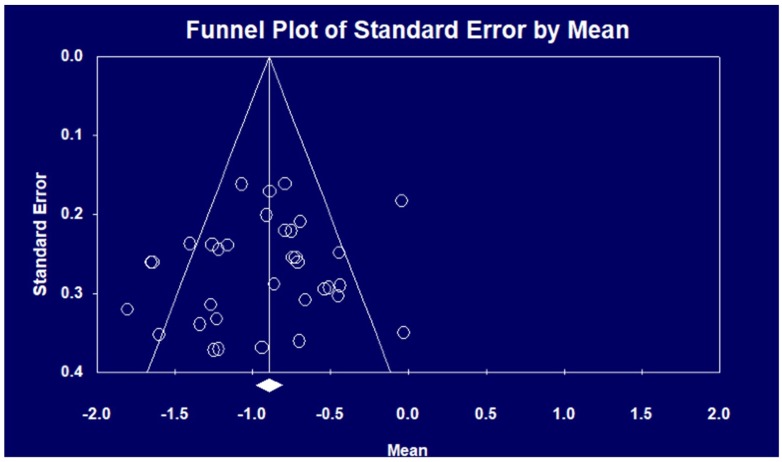
Funnel plot of the selected studies.

**Figure 3:**
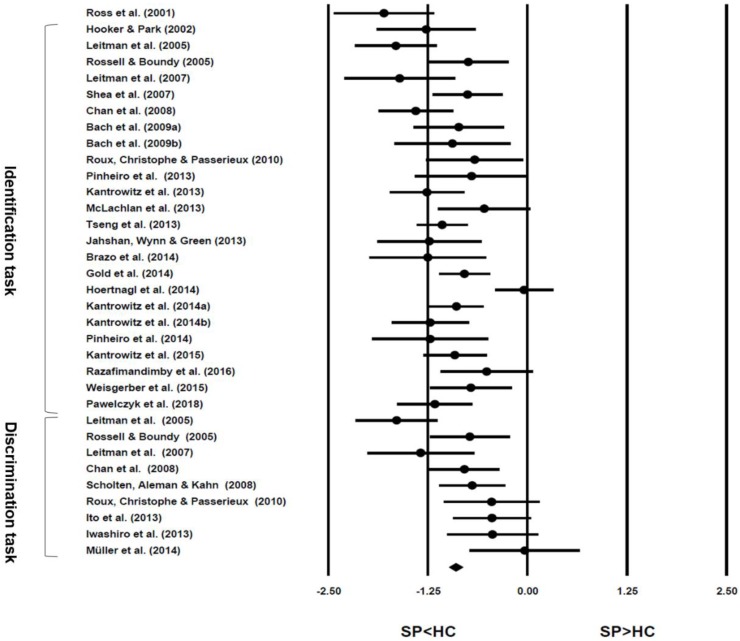
Forest plot with effect size (*d*) and confidence intervals for the selected studies. Note: Ross et al.’s study (2001) only reported the results of comprehension tasks, thus excluded from the meta-analysis of both identification and discrimination paradigm but included in the overall effect size calculation.

All studies considered accuracy as the indicator of participants’ behavioral performance of EPP. Five studies also recorded the response time for between group comparisons [35,37,38,41,55]. Among the studies with healthy participants as a control group, nearly all studies (27/29) reached a consensus that schizophrenia patients (at least one of the schizophrenic patient groups) showed poorer performance than healthy controls except one study that tested audio-visual emotional integration [50] and another involving symptomatically remitted schizophrenic patients [11]. These two studies corresponded to the outliers in the right side of the funnel plot in Figure 2 with the mean value standing at 0, indicating no significant difference between patients and healthy control in EPP performance. Analysis of schizophrenic patients’ EPP impairment across the entire studies revealed a large overall effect size: *d* = −0.92, 95% CI = −1.06 < δ < −0.78. The effect size was larger for identification test paradigm: *d* = −0.95, 95% CI = −1.11 < δ < −0.80, but smaller for discrimination tasks (*d* = −0.74, 95% CI = −1.03 < δ < −0.44). It is worth noticing that there was a study that only reported the results of comprehension tasks; thus, it was excluded from both identification and discrimination paradigm but included in the overall effect size calculation [28]. The overall meta-analysis results are presented in a funnel plot (Figure 2) and a forest plot (Figure 3).

There remained some inconsistent findings among studies dealing with different subtypes of schizophrenia (e.g., SAVH vs. SNAVH), the influence of which will be explained in the next section. Studies involving patients with other diseases suggested that patients with schizophrenia performed worse than those with depression [36], and their EPP were more statistically similar to RBD patients than LBD patients [28].

Less than half of the studies took recognition of single emotion into consideration, and even fewer studies analyzed the significant differences between groups from this perspective. The seven studies with between-group performance of single and overall emotion recognition are demonstrated in Table 2 and Table 3. Although between-group recognition performance varied from one type of emotion to another, the most consistent result was the evidence of schizophrenic patients’ more impairment in recognizing emotional stimuli than neutral ones [31,37,41,47].

Two studies further explored the misclassification of emotions during EPP tasks. Patients with auditory verbal hallucination were found to be more likely to misclassify happiness into fear and fear into sadness [31], while another study found that they were more likely to mislabel sadness into happiness [33]. However, Shea and his colleagues further pinpointed that patients without auditory verbal hallucination tended to misidentify sadness as neutrality, again indicating the differences associated with subtypes of schizophrenia.

### 3.3. Influential Factors of Emotional Prosody Processing

The major influential factors that were reported by the selected studies can be categorized into two types: factors related to participants and factors related to experimental design.

#### 3.3.1. Factors Related to Participants

##### Demographic Factors

Among the major demographic factors, selected studies found no differences that were attributable to IQ or education [33,38]. As for gender, a non-significant effect was identified in some studies [30,36]. However, female patients were found to preserve their advantages in EPP in another experiment [35], which explained the reason why social functioning in schizophrenic patients was less impaired in women than men.

##### Cognitive Factors

A series of studies delved into the cognitive factors influencing schizophrenic patients’ EPP performance. Abnormal sensory processing, especially basic auditory processing of pitch and intensity, were significantly correlated with deficits in EPP [30,42,45,53]. Also, though it was claimed that no influence was exerted by attention in one study [33], more studies recognized both pre-attentive and attentive processing as important predictors of emotion recognition tasks [34,41,42,53].

##### Clinical Factors

Using PANSS, several studies identified the negative correlation between the severity of schizophrenic illness and EPP performance [38,46,50], but few studies discovered any difference made by the duration of diseases [33].

Positive symptom scores were found to contribute to a positive correlation with emotions of positive valence by more scholars [40,41,46] with the exception of one study reporting no correlation [36]. Three of the selected studies also examined the effects of specific positive symptoms such as hallucination and delusion, which generally exacerbated schizophrenic patients’ impairment in EPP [33,41,45]. However, this finding failed to be replicated when hallucinating patients were asked to identify the emotional prosody of semantically neutral sentences [44], indicating that EPP of schizophrenic patients was a rather complex process influenced by more than one factor.

Compared with positive symptoms, negative symptoms such as anhedonia were more consistently recognized to demonstrate a strong correlation with patient’s deficits in EPP. Such a correlation was discovered with the aid of both behavioral and neurological research techniques [30,35,36,38,43,46].

#### 3.3.2. Factors Related to Experiments

##### Task-Related Factors

A small number of studies explored the effects of task types in schizophrenic patients’ EPP [38]. The major finding was that compared with normal participants, schizophrenic patients had more difficulty in identification tasks than discrimination ones, and identifying the emotional prosody of the stimuli was even harder than emotional content [38].

Apart from emotional prosody tests, there were also studies (10 out of 29) including emotion recognition tasks in other modalities. This is not surprising as face-to-face interpersonal communication is inherently multimodal with both verbal and nonverbal signals, including tone of voice, facial expressions, hand gestures and body movements. However, the relationship among visual, vocal and musical modalities of test was still controversial. While facial expression identification was referred as a satisfactory predictor of emotional prosody in one study [36], more scholars discovered the disassociated processing of different modalities [29,30,34].

##### Stimulus-Related Factors

Among the stimulus-related factors, the emotional valence of stimuli had received most attention of the selected research. As suggested in the previous section, studies consistently found that patients with schizophrenia were more impaired in processing emotional stimuli than neutral ones [31,37,40,41,50,51]. It was further indicated that sadness was most difficult to detect for patients among all the emotions [35,47].

The presence of semantic information, the congruency of emotional valence and the clarity of emotion were also proved to be influential factors of schizophrenic patient’s EPP performance. Patients improved their performance when stimuli with semantic information were presented [51], and they showed an even better performance when semantic content and affective prosody of the stimuli shared a congruent emotional valence [47,50]. By contrast, patients were more likely to make an erroneous interpretation if they were asked to process low-clarity or potentially ambiguous emotions [29].

### 3.4. Neural Mechanisms of Emotional Prosody Processing

#### 3.4.1. Hemispheric Processing of Emotional Prosody

Right hemisphere had been repeatedly reported dominant in EP processing for healthy controls [63,64]. The deficits in EPP suggested that the right hemispheric processing was disturbed for schizophrenic patients [28,57]. The right-lateralization decreased in patients with stronger negative symptoms and with hallucinating symptoms [37]. There was also a study suggesting left-lateralized abnormalities in schizophrenic patients, but it lacked neuroimaging data to support its conclusion [41].

#### 3.4.2. Spatial Localization of Brain Networks for Processing Emotional Prosody

Three studies identified structural and functional disturbances of brain regions associated with the deficits in processing emotional prosody for schizophrenic patients by means of MRI tools. It was revealed that there might be dysfunctions in (a) primary auditory cortex [32] and (b) medial prefrontal cortex [55], both of which were important for social communication deficits. It was also identified reduced auditory-insula connectivity as a determinant of social cognitive dysfunction in schizophrenic patients [53].

#### 3.4.3. Temporal Processing of Emotional Prosody

The neural correlates for examining the time course of EPP in the selected ERP studies included mismatched negativity (MMN), P50, P1, N100, P200 and P300. Significant reduction in MMN indicated an impaired pre-attentive processing in schizophrenia [42,53]. A reduced P300 and a significant correlation between MMN and P300 within the patient group were found, which demonstrated that pre-attentive (MMN) and later attention-dependent processes (P300) contributed generally equally to EP change detection in patients with schizophrenia [42].

Other neural correlates investigated in the selected studies were mainly associated with sensory processing of the emotional valence of the stimuli. For example, there were also reduced P1, P50 and N100 when patients were processing emotionally incongruent stimuli, happy stimuli and neutral stimuli respectively [45,50,51]. However, increased P200 was observed when happy stimuli were presented, which was correlated with delusions [45,51].

## 4. Discussion

Overall, despite differences across studies in the current selective review, there is a general pattern of significantly worse performance of EPP in schizophrenic patients compared with healthy controls with large effect sizes as shown in our meta-analysis, which is consistent with the previous review [24]. Our analysis further revealed a more severe impairment in perceiving EP in identification tasks. Moreover, the studies consistently found a significant association between EPP and participants’ cognitive and clinical factors such as auditory processing deficits, the severity and negative symptoms of the disease. The cognitive brain research studies further demonstrated that schizophrenic patients had EPP dysfunctions in both pre-attentive and attentive cognitive processes, which can be attributed to disorders in right hemisphere and brain regions such as medial prefrontal cortex, primary auditory cortex and auditory-insula connectivity. However, some of the findings involving stimuli of different modalities, participants of different genders, language backgrounds and subtypes as well as positive symptoms of schizophrenia, remained controversial. The convergent and divergent findings have important implications for clinical practice and research.

### 4.1. Implications of the Selected Studies

#### 4.1.1. Implications for Practice

One of the most salient findings from this review, is that schizophrenic patients generally had poorer performance in processing emotional prosody. To date, emotional prosody has been recognized as an important window to detect schizophrenic deficits, and it has also played a role in improving people’s social communication [47,50]. The feasibility and efficacy of EPP intervention have been demonstrated in several studies. For example, a recent systematic review and meta-analysis concluded that future clinical trials should consider applying a cognition remediation program that combines social cognition training elements and psychiatric rehabilitation [14]. An empirical study also demonstrated that schizophrenic patients were able to improve their affective prosody perception while being involved in community-based psychosocial rehabilitation programs [13]. Cognitive ability such as melodic discrimination and the factors influencing cognition such as acoustic features of the stimuli were also valued as crucial targets of cognitive remediation in schizophrenia [39,49]. However, to our knowledge, few programs have incorporated it into the early diagnosis and remediation of schizophrenic disorders [38]. Current clinical classifications including both the revised version of the WHO International Classification of Disorders (ICD-11) and the Diagnostic and Statistical Manual of Mental Disorders (DSM-5) by the American Psychiatric Association also did not specify EPP as a target area for assessment or treatment [65,66], although the DSM-5 classification system does include functional impairment and the ICD-11 encourages the use of functional impairment as diagnostic criteria. The strong effect sizes across the reviewed studies provide the most telling evidence that emotional prosody, either in verbal or non-verbal forms (e.g., natural sounds, music), should be implemented as an important supplemental clinical indicator in the early detection of schizophrenia. For example, it is possible to better distinguish patients with schizophrenia from healthy controls by observing their EP performance in perceptual tasks with emotional stimuli of different modalities and in identification paradigms. Furthermore, a milder approach could be combined with clinical medication in the rehabilitation of schizophrenia. For example, a line of studies pinpointed the role of bottom-up cognitive remediation paradigms [42,43,53]. Apart from sensory-cognitive methods, it is advisable to consider employment of other behavioral therapeutic approaches of psychology and speech pathology, in which EP can be an important component.

#### 4.1.2. Implications for Research

There are some apparent limitations of the selected studies. First, none of the included research works were longitudinal studies, which failed to provide data tracing patients’ performance over time. Schizophrenia is known to be a psychiatric disorder that varies in severity with a time course of single or multiple psychotic episodes potentially leading to life-long disability. The short-term and long-term characteristics in symptomology may show different trajectories of relapses and remissions for positive and negative symptoms along with the treatment time course and medication history. Similarly, cognitive functioning such as executive functions, attentional control, and working memory is known to show age-related differences and changes. However, none of the studies covered in the current review delved into symptomology changes and changes in overall cognition in relation to the patients’ EPP ability. Second, only two of the 29 studies addressed the production of schizophrenic patients’ emotional prosody so that production and perception data could not be directly compared across the studies. As difficulties in distinguishing self and other is at the core of schizophrenia, it remains an important question for researchers to explore the exact forms of relationship between EP comprehension and production in these patients. Third, the number of studies conducted in different languages was imbalanced, with non-tonal languages outnumbering tonal ones and stressed-timed languages outnumbering syllable-timed ones.

More cross-sectional and longitudinal studies are needed to investigate the behavioral and neuro-cognitive mechanisms of schizophrenic patients’ perception and production of emotional prosody. There are at least three new directions for future studies in this regard.

First, more studies conducted in tonal languages (e.g., Mandarin Chinese) and syllable-timed languages (e.g., Mandarin Chinese and French) are called for in this field [34]. More importantly, there needs to be more cross-linguistic investigation of the relationship between emotional prosody and linguistic units extending from phonetics and semantics to morphology, grammar, syntax and pragmatics. The process of verbal communication may vary from not only speaker to speaker, but also from language to language. The selected studies did not employ the same tasks to compare patients with different ethnic, language and cultural backgrounds, and to associate EP perception with their EP production, thus rendering the cross-language generalization difficult. Moreover, as an important component of extralinguistic domains, prosody is closely connected with other linguistic domains in understanding speaker’s meaning. Thus, new directions for research will surely benefit from new perspectives such as schizophrenic patients’ cross-linguistic and cross-cultural communication differences in EP perception and production.

Secondly, given the existing controversial views about emotional recognition of different modalities, the influences of different sensory domains are worthy of further exploration. Currently, a line of studies has discovered schizophrenic patients’ deficits in processing emotional stimuli not only in vocal modalities but also in visual forms [67]. The disassociation among different modalities in understanding emotions was also found [29,30,34]. However, the reasons for the disassociated results may lie in that these studies tended to separate cross-modal processing of emotion in different tasks rather than in the same one, in which visual and vocal stimuli were presented simultaneously [50]. By contrast, studies of face-voice emotion integration in normal subjects have shown strong facilitation effects in terms of accuracy, sensitivity, and reaction time [68]. De Silva et al. demonstrated that in normal subjects, some emotions such as sadness and fear in videos are better identified in the auditory modality whereas other emotions such as anger and happiness are better recognized in the visual modality [69]. A recent EEG study on normal subjects further revealed visual-auditory priming effects in distinct neural oscillatory activities for emotional prosody processing as against phonetic processing [70]. It remains to be tested how patients with schizophrenia differ from normal individuals in such multimodal experimental paradigms. Only by involving different forms of stimuli (such as visual, auditory, and tactile information) in the same task can we truly test the interactions in multisensory integration, which is required of daily functions in face-to-face social interactions.

Finally, cognition also serves as a promising testbed for researching schizophrenic patients’ comprehension of emotional prosody. It has been discovered that both pre-attentive and attentive processing play significant roles in emotion recognition tasks [34,41,42,53]. Future studies concerning emotional prosody understanding can be extended to other neuro-cognitive domains such as memory, monitory, thoughts and reasoning, literacy, language production and problem-solving abilities. It remains to be explored how EPP deficits may be linked with genetics, brain chemistry and specific brain circuits (or information processing pathways), neuroanatomical abnormality, and environmental factors. Furthermore, as EPP deficits were identified in other psychotic disorders such as borderline personality disorder [16] and autism spectrum disorder [17], similarities and differences in behaviors, neurocognitive structures and functions, and genetic representations among different types of patients deserve in-depth investigation, which may lay a more solid foundation for effective intervention of schizophrenia and for the exploration of brain plasticity in future research [71]. Despite the similarities on behavioral and psychological measures of social cognition deficits that may be shared across different types of clinical patients including schizophrenia, it remains to be investigated whether their behavioral symptomology may reveal subtle distinctions and how those can be traced down and linked with distinct underlying neural mechanisms and genetic epidemiology.

### 4.2. Limitations of This Selective Review

Our own analysis in this review bears several limitations. First, our review was limited to peer-reviewed publications which were hypothesis-driven research studies, and because of the large quantity of studies during the searching process, there might be a few missing reports even though we strived to be careful when including the studies. Second, as the sample size and comprehensiveness of neuropsychological profiles of the patients varied widely in the studies, caution is necessary to interpret the results from different subtypes of schizophrenic patients, different task paradigms and response patterns, and stimuli of different modalities, emotional valence and presentation forms. The breadth of types of studies may also pose obstacles to generalizing some of the results. Due to space consideration, our classification of identification vs. discrimination tasks and characterization of the materials and details of the experimental protocols are likely oversimplified in the summary description. Finally, the selection criteria could serve as a potential source of bias in estimating the overall significance of EPP deficits in schizophrenic patients. In our search and selection criteria, we excluded patients with co-morbidity and prodromal schizophrenia. We originally intended to narrow down the scope of participants and provide a more accurate description of patients who only suffered from schizophrenia at the time of the experiments, but this may, to some extent, render our selective review disconnected from the real-world diagnosis, prevention, and remediation of schizophrenia. In this regard, some recent studies have touched upon schizophrenic patients at various stages of the disease such as prodromal schizophrenia [52], first-episode schizophrenia [56], and chronic schizophrenia [48] as well as with different co-morbid disorders such as depression [18,56] and schizoaffective disorders [19], which may be enlightening for future empirical research.

## 5. Conclusions

The present study provides a selective review of the literature on emotional prosody processing in schizophrenic patients. A total of 29 behavioral and neural studies with great variability in research topics, experimental design and study participants were selected and summarized. Compared with healthy controls, schizophrenic patients showed worse performance of EPP, especially in identification tasks involving emotional and neutral stimuli than in discrimination tasks. Apart from experiment-related factors, the EPP deficits were generally found to be associated with cognitive and clinical factors such as auditory processing deficits, the severity and negative symptoms of the disease, while demographic factors such as IQ and education proved to make little difference. Neural evidence indicated impairments in patients’ right hemisphere and dysfunctions in primary auditory cortex, medial prefrontal cortex, and auditory-insula connectivity, which provided a biological explanation for their social cognitive dysfunction. Moreover, the patients showed impaired pre-attentive and attentive processes, both of which played important roles in their EPP. The current review supports the use of EP assessment in early diagnosis and rehabilitation of schizophrenic disorders in clinical practice. Future cross-sectional and longitudinal study topics are further suggested to gain insights about schizophrenic patient’s perception and production of EP in different languages and cultures, modality forms and neuro-cognitive domains.

## Figures and Tables

**Table 1:** Characteristics of studies related to emotional prosody processing (EPP) in schizophrenia.

Year	First Author	Study Type (Neurological Technique): Study Topic	Country Language	Task Paradigm	Stimulus Modality: Stimulus Form	Response Option	Patient Information					Statistical Analysis Methods	Notes (Reasons for Exclusion from Meta-Analysis)
							Number (Male%)	Age (S.D.)	Education (years)	IQ	Illness Duration (years)		
2001	Ross [28]	Behavioral: Hemispheric dysfunction in schizophrenia patients and the neurology of EPP	the US English	Identification (SSC and SPP) Discrimination (SSC pair)	A: sentences, monosyllables, words and asyllabic sounds	C	45 (87%)	42.5 (7.2)	13.8 (2.3)	N/A	N/A	ANOVA, Chi-square test and PCA	Excluded from the meta-analysis of identification and discrimination task but included in the overall effect size calculation (only reporting the results for comprehension)
2002	Hooker [29]	Behavioral: EPP and social functioning	the US English	Identification (SSC)	A: sentences, V: facial pictures	C	20 (75%)	39.3 (8.5)	12.7 (2.9)	N/A	18.8 (10.2)	ANOVA	
2005	Leitman [30]	Behavioral: Impaired EPP and basic auditory processing deficits	the US English	Identification (SSC) Discrimination (SSC pair)	A: sentences, V: facial pictures	C	43 (77%)	39.0 (12.0)	10.6 (3.2)	N/A	17.4 (9.6)	ANOVA, Spearman and PCA	
2005	Rossell [31]	Behavioral: Impaired EPP and auditory-verbal hallucinations	Australia English	Identification (SSC) Discrimination (SSC pair)	A: sentences, words, non-words, syllables V: pictures	R and C	40 (60%) AVH:20	41.5 (9.5)	13.9 (2.8)	104.0 (14.7)	23.9 (20.4)	ANOVA and SNK	
							NAVH: 20	36.9 (8.9)	14.6 (3.7)	104.3 (12.6)	15.7 (20.6)		
2007	Leitman [32]	Behavioral and neural (MRI): Neural substrates of impaired EPP	the US English	Identification (SSC) Discrimination (SSC pair)	A: sentences	C	24 (88%)	32.5 (10.6)	11.0 (2.0)	94.1 (7.5)	N/A	ANOVA, Spearman, voxel wise correlation approach and PCA	
2007	Shea [33]	Behavioral: EPP and auditory hallucinations	Australia English	Identification (SSC) Discrimination (SSC pair)	A: sentences	C	67 (69%) AH:38	40.0 (10.0)	14.0 (3.0)	108.0 (11.0)	14.0 (9.0)	ANOVA and Tukey‘s HSD	
							NAH:29	44.0 (11.0)	14 (3)	109.0 (11.0)	20.0 (15.0)		
2008	Chan [34]	Behavioral: EPP and neuropsychological function	China (HK) Chinese	Identification (SSC) Discrimination (SSC pair)	A: sentences (meaningless) V: facial photos	C	43 (49%) PS:19	34.5 (9.3)	11.7 (2.1)	N/A	8.9 (7.9)	MANOVA and Multiple stepwise linear regression	
							NPS:24	40.0 (9.1)	8.8 (3.4)				
2008	Scholten [35]	Behavioral: Gender differences and IQ in EPP	The Netherlands Dutch	Discrimination (meaning-prosody stroop test)	A: sentences	C	48 (52%)	M: 29.5 (7.0)	N/A	M: 107.2 (9.6)	7.0 (4.9)	ANOVA, ANCOVA and Pearson	
								F: 32.2 (6.6)		F: 110.2 (8.3)	8.5 (6.6)		
2009a	Bach [36]	Behavioral: High clarity and EPP	Switzerland German	Identification (SPP)	A: sentences (meaningless) V: facial photos	C	25 (52%)	35.9 (11.8)	N/A	N/A	6 (N/A)	ANOVA	
2009b	Bach [37]	Behavioral and neural (fMRI): Lateralization of EPP	Switzerland German	Identification (SPP)	A: non-words	C	15 (53%)	31.6 (7.9)	N/A	N/A	N/A	ANOVA, ANCOVA, SPM and laterality measures	
2010	Roux [38]	Behavioral: Implicit and explicit of EPP	France French	Identification (SSC) Discrimination (meaning-prosody stroop test)	A: words	C	21 (67%)	38.2 (12.6)	11.5 (2.9)	103.7 (7.0)	N/A	ANCOVA, ANOVA and Pearson	
2012	Gold [39]	Behavioral: The relationship between auditory emotion recognition impairments and acoustic features and cognition	the US English	Identification (SPP)	A: vocal sounds (full version and brief version which contains intensity modulation and pitch modulation) V: facial expressions	C	92 (86%)	37.8 (10.4)	N/A	N/A	N/A	ANOVA, multivariate regression and path analysis	Identification of brief version of vocal sounds excluded from meta-analysis (task quite different from other studies’)
2013	Ito [40]	Behavioral: EPP and positive psychotic symptoms	Japan Japanese	Discrimination (meaning-prosody stroop test)	A: sentences	C	28 (61%)	30.9 (8.1)	13.6 (2.0)	N/A	6.9 (7.3)	ANOVA, *T* test and Spearman	
2013	Iwashiro [41]	Behavioral: Semantic processing of emotional content and auditory attention	Japan Japanese	Discrimination (dichotic listening of SSC pair)	A: words	C	22 (50%)	31.6 (5.2)	13.5 (1.8)	96.7 (10.0)	8.7 (6.7)	ANOVA, Paired-samples *T* test and Spearman	
2013	Jahshan [42]	Behavioral and neural study (ERP): Auditory processing and EPP	the US English	Identification (SSC)	A: sentences V: photos of facial expressions	C	36 (69%)	47.7 (10.0)	12.6 (1.8)	N/A	24.3 (11.5)	Independent-samples *T* test, Chi-square test, One-sample *T* test, MANOVA, Pearson and Multiple regression analysis	
2013	Kantrow-itz [43]	Behavioral: Emotion recognition based on tone of voice and basic auditory processing	the US English	Identification (SSC and SPP)	A: FM tones and sentences	C and R	41 (53%)	36.5 (10.9)	12.3 (2.3)	N/A	16.0 (10.0)	ANOVA, Independent-samples *T* test, M-W test, multivariate linear regression and Pearson	
2013	McLach-lan [44]	Behavioral: EPP and auditory hallucinations	Australia English	Identification (SSC)	A: sentences	R	34 (76%) AVH:19	41.2 (9.9)	13.2 (2.5)	104.2 (8.8)	N/A	ANOVA, Chi-square test and Independent-samples *T* test	
							NAVH:15	43.5 (8.8)	12.9 (2.6)	101.2 (9.7)			
2013	Pinheiro [45]	Behavioral and neural (ERP): ERP correlates of EPP	the US English	Identification (SSC and SPP)	A: sentences and sentences (meaningless)	C	15 (100%)	49.7 (9.1)	14.33 (1.8)	92.2 (29.4)	22.3 (10.5)	MANOVA and Spearman	
2013	Tseng [46]	Behavioral: EPP across modalities and psychotic symptoms	China (Taiwan) Chinese	Identification (SPP)	A: sounds V: facial photos	C	111 (46%)	38.2 (10.1)	15.9 (3.4)	92.5 (16.3)	13.8 (9.7)	Independent-samples *T* test, Chi-square test, ANCOVA, Pearson, Multiple bidirectional stepwise linear regression, and Multiple linear regression	
2014	Brazo [47]	Behavioral: EP comprehension and semantic content	France French	Semantic identification (EPP influences)	A: sentences	C	16 (56%)	39.7 (8.6)	N/A	89.8 (11.5)	13.3 (5.8)	ANOVA, ANCOVA and Paired-samples *T* test	
2014	Dondai-ne [48]	Behavioral: facial and vocal emotion recognition biases	France French	Emotion intensity rating	A: non-verbal bursts without semantic content (vowel “ah”) V: facial expressions	R	23 (65%)	33.9 (7.3)	12.7 (2.0)	N/A	12.4 (6.5)	ANOVA, independent sample *T* test, Spearman’s correlation analysis	The whole study excluded from meta-analysis (task quite different from other studies’)
2014	Hoertna-gl [11]	Behavioral: A comparison of EPP between symptomatically remitted patients with schizophrenia and bipolar disorder	Austria N/A	Identification (SSC)	A: sentences	C	41 (54%)	40.5 (8.5)	12.9 (2.9)	N/A	12.4 (6.9)	Chi-square test, ANOVA, multiple linear regression	
2014a	Kantrow-itz [19]	Behavioral and neural (MRI): early sensory processing and sarcasm perception	the US English	Identification (SSC) Discrimination (sarcasm perception)	A: sentences	C	76 (63%)	37.4 (10.1)	12.1 (2.3) (74 patients)	N/A	15.3 (9.0) (74 patients)	ANOVA, independent sample *T* test, Pearson correlations and multivariate linear regression	Discrimination task excluded from meta-analysis (sarcasm is a complex social emotion)
2014b	Kantrow-itz [49]	Behavioral: amusia and protolanguage impairments in schizophrenia	the US English	Identification (emotional categories and intensity of SSC)	A: phrases	R	31 (87%)	39.4 (9.9)	11.4 (2.2) (29 patients)	N/A	14.8 (8.2) (24 patients)	independent-samples *T* test, MANOVA, ANOVA, multivariate linear regression and Pearson	Identification of emotional intensity excluded from meta-analysis (tasks quite different from other studies’)
2014	Müller [50]	Behavioral and neural study (ERP): Neural substrates of auditory emotion recognition deficits	the US English	Discrimination (face-prosody stroop test)	A and V: sounds and pictures of faces	R	15 (73%)	35.1 (9.3)	14.1 (2.2)	N/A	14.3 (9.1)	ANOVA, MANOVA, Bonferroni correction and Pearson	
2014	Pinheiro [51]	Behavioral and neural study (ERP): ERP correlates of EPP	the US English	Identification (SSC and SPP)	A: words and non-words	C	16 (69%)	48.9 (7.4)	14.0 (2.4)	>85	19.5 (11.0)	ANOVA	
2015	Corcora-n [52]	Behavioral: emotion recognition deficits as predictors of transition in individual at clinical high risk for schizophrenia	the US English	Identification (SSC)	A: sentences V: facial expressions	C	7 (57%)	20.0 (5.2)	N/A	N/A	N/A	ANOVA	The whole study excluded from meta-analysis (involving prodromal patients)
2015	Kantrow-itz [53]	Behavioral and neural study (ERP and rsfMRI): Neural substrates of auditory emotion recognition deficits	the US English	Identification (SSC and SPP)	A: FM tones and sentences	C	84 (81%)	39.4 (10.6)	N/A	N/A	15.9 (9.4)	ANOVA, MANOVA, Independent-samples *T* test, multivariate linear regression, Pearson and SPM	
2015	Regenbo-gen [18]	Behavioral and neural (fMRI): Neural responses to multimodal stimuli and pathology-specific social cognition deficits	Germany German	Empathy rating	A and V: video clips expressing emotion through three channels: facial expression, prosody and content	R	20 (N/A)	37.3 (8.4)	N/A	N/A	N/A	ANOVA, Pearson correlation analysis and SPM	The whole study excluded from meta-analysis (involving participants’ empathy, which is a complex social emotion)
2015	Sterea [9]	Behavioral: the relationship between social cognition and functional outcomes in schizophrenia	Romania Romanian	Definition of emotion and explanation of emotional situations and events	N/A	interview	15 (60%)	41.9 (8.4)	N/A	N/A	N/A	M-U test and Kendall correlation	The whole study excluded from meta-analysis (task quite different from other studies’ and involving complex emotions such as surprise and suspiciousness)
2015	Weisger-ber [54]	Behavioral: Facial, vocal and musical emotion recognition	Belgium French	Identification (SPP)	A: non-verbal vocal affect bursts V: computer-generated faces	R	30 (37%)	35.5 (12.7)	12 (2.2)	N/A	10.9 (9.4)	ANOVA, MANOVA and Spearman	
2016	Razafim-andimby [55]	Behavioral and neural study (fMRI): Neural bases of emotional sentence attribution	France French	Identification (SSC)	A: sentences	C	21 (76%)	33.9 (7.4)	N/A	N/A	11.9 (7.9)	MANOVA, Chi-square test, SPM and Wilcoxon Rank Test	
2017	Hernim-an [56]	Secondary analysis: the effect of comorbid depression on facial and prosody emotion recognition	Australia English	N/A	N/A	N/A	82 (65.9%)	21.1 (2.6)	N/A	93.3 (13.2)	N/A	ANCOVA and partial correlation analysis	The whole study excluded from meta-analysis (involving participants with comorbid disorders and secondary analysis of the data)
2018	Pawełcz-yk [57]	Behavioral: Extralinguistic and paralinguistic processing meditated by right hemisphere	Poland Polish	Identification (SPP)	A: sentences (meaningless)	C	40 (58%)	26.3 (9.3)	12.0 (2.6)	N/A	3.9 (4.7)	*T* test, Chi-square Test and M-U test	

Note. (1) Abbreviations: fMRI = functional magnetic resonance imaging; ERP = event-related potential; rsfMRI = resting state functional magnetic resonance imaging; SSC = stimuli with semantic content; SPP = stimuli with pure prosody (without semantic content); FM tone = frequency-modulated tone; C = choice; R = rating; A = auditory; V = visual; A and V = auditory and visual stimuli simultaneously appearing in the same task; AVH = auditory verbal hallucination; NAVH = non-auditory verbal hallucination; M = male; F = female; ANOVA = Analysis of Variance; ANCOVA = Analysis of Covariance; MANOVA = Multivariate Analysis of Variance; M-U = Mann–Whitney U test; SNK = Student–Newman–Keuls test; PCA = Principal Component Analysis; SPM = Statistical Parametric Mapping; N/A = not available. (2) Patients’ information involves all the patients who participated in the corresponding studies regardless of different task paradigms. (3) For 42.5 (7.2), the mean is 42.5 and S.D. is 7.2.

**Table 2:** EPP performance of participant groups (Identification task).

Year	First Author	Effect Size of Performance in Schizophrenic Patients as Compared to Healthy Control (95% CI)	Task Paradigm and Material	Type of Participants (Number)	Single Emotion Recognition						Overall Emotion Recognition	
					Happy	Sad	Angry	Fearful	Surprised	Disgusted	Neutral	
2002	Hooker	*d* = −1.27 (−1.91 to −0.64)	Identification (SSC)	SP (20)-HC (27)								▲
2005	Leitman	*d* = −1.65 (−2.17 to −1.13)	Identification (SSC)	SP (43)-HC (34)								▲
2005	Rossell	*d* = −0.74 (−1.25 to −0.23)	Identification 1 (SSC)	SAVH (20)-HC (26)	●	▲	●	▲				▲
				SNAVH (20)-HC (26)	●	●	●	▲				▲
				SAVH (20)-SNAVH (20)	●	▲	●	●				▲
			Identification 2 (SSC)	SAVH (20)-HC (26)	●	▲	▲		▲		●	▲
				SNAVH (20)-HC (26)	●	▲	▲		▲		●	▲
				SAVH (20)-SNAVH (20)	●	●	▲		●		●	●
2007	Leitman	*d* = −1.60 (−2.32 to −0.89)	Identification (SSC)	SP (24)-HC (17)								▲
2007	Shea	*d* = −0.75 (−1.18 to −0.31)	Identification (SSC)	SAH (38)-HC (31)	●	●					●	▲
				SAH (38)-SNAH (29)	●	●					●	▲
				SNAH (29)-HC (31)	●	●					●	●
2008	Chan	*d* = −1.40 (−1.87 to −0.93)	Identification (SSC)	SP (43)-HC (43)								▲
2009a	Bach	*d* = −0.86 (−1.44 to -0.28)	Identification (SPP)	SP (25)-HC (25)	●	●	●	●		●	●	▲
				SP (25)-Depression (25)	●	●	●	▲		●	●	▲
2009b	Bach	*d* = −0.94 (−1.69 to −0.18)	Identification (SPP)	SP (15)-HC (15)								▲
2010	Roux	*d* = −0.66 (−1.28 to −0.04)	Identification (SSC)	SP (21)-HC (21)								▲
2012	Gold	*d* = −0.79 (−1.11 to −0.47)	Identification (SPP-full version)	SP (92)-HC (73)	●	▲	●	▲		▲	▲	▲
2013	Jahshan	*d* = −1.23 (−1.90 to −0.56)	Identification (SSC)	SP (34)-HC (14)	●	●	●	●		●	●	▲
2013	Kantrowitz	*d* = −1.26 (−1.73 to −0.79)	Identification (SSC and SPP)	SP (41)-HC (41)								▲
2013	McLachlan	*d* = −0.54 (−1.13 to 0.06)	Identification (SSC)	SP (34)-HC (17)								▲
2013	Pinheiro	*d* = −0.70 (−1.44 to 0.04)	Identification (SSC)	SP (15)-HC (15)	●		▲				●	▲
			Identification (SPP)	SP (15)-HC (15)	●		●				▲	▲
2013	Tseng	*d* = −1.07 (−1.39 to −0.75)	Identification (SPP)	SP (111)-HC (70)								▲
2014	Brazo	*d* = −1.25 (−2.01 to −0.49)	Semantic identification (EPP influences)	SP (16)-HC (16)								▲
2014	Hoertnagl		Identification (SSC)	SP (41)-BD (58)	●	●	●	●				●
		*d* = −0.04 (−0.41 to 0.33)	Identification (SSC)	SP (41)-HC (85)	●	●	▲	●				●
2014a	Kantrowitz	*d* = −0.89 (−1.23 to −0.55)	Identification (SSC)	SP (76)-HC (72)								▲
2014b	Kantrowitz	*d* = −1.22 (−1.72 to −0.72)	Identification (emotional categories of SSC)	SP (31)-HC (44)								▲
2014	Pinheiro	*d* = −1.22 (−1.98 to −0.47)	Identification (SSC)	SP (16)-HC (16)	●		▲				●	●
			Identification (SPP)	SP (16)-HC (16)	▲		●				●	●
2015	Kantrowitz	*d* = −0.91 (−1.31 to −0.51)	Identification (SSC and SPP)	SP (58)-HC (49)								▲
2015	Weisgerber	*d* = −0.71 (−1.23 to −0.19)	Identification (SPP)	SP (30)-HC (30)	●	●	●	●	●	●	●	▲
2016	Razafimandimby	*d* = −0.51 (−1.10 to 0.08)	Identification (SSC)	SP (21)-HC (25)								
2018	Pawełczyk	*d* = −1.16 (−1.63 to −0.68)	Identification (SPP)	SP (40)-HC (39)								▲

Note. (1) Negative effect size Cohen’s d indicates worse performance in schizophrenic patients compared to healthy control; (2) Twenty-four out of 29 studies are reported in this table. Other studies are not reported since they did not employ identification paradigm; (3) SSC = stimulus with semantic content; SPP = stimulus with pure prosody (without semantic content); SP = schizophrenic patients; SAVH = schizophrenia with auditory verbal hallucination; SAH = schizophrenia with auditory hallucination; SNAH = schizophrenia without auditory hallucination; SNAVH = schizophrenia without auditory verbal hallucination; HC = healthy control; BD = patients with bipolar disorder; ▲ = Significant difference (*p* < 0.05); ● = non-significant difference; blank = not evaluated; (4) The data were collected based on the accuracy rate reported in the included studies.

**Table 3:** EPP performance of participant groups (Discrimination task).

Year	First Author	Effect Size of Performance in Schizophrenic Patients as Compared to Healthy Control (95% CI)	Task Paradigm and Material	Type of Participants (Number)	Single Emotion Recognition							Overall Emotion Recognition
					Happy	Sad	Angry	Fearful	Surprised	Disgusted	Neutral	
2005	Leitman	*d* = −1.64 (−2.16 to −1.12)	Discrimination (SSC pair)	SP (43)-HC (34)								▲
2005	Rossell	*d* = −0.72 (−1.23 to −0.21)	Discrimination (dichotic listening of SPP pair)	SAVH (20)-HC (26)	▲	●	●	▲			●	▲
				SNAVH (20)-HC (26)	●	●	●	●			●	●
				SAVH (20)-SNAVH (20)	▲	●	●	▲			●	▲
2007	Leitman	*d* = − 1.34 (−2.03 to −0.65)	Discrimination (SSC pair)	SP (24)-HC (17)								▲
2008	Chan	*d* = −0.79 (−1.23 to −0.35)	Discrimination (SSC pair)	SP (43)-HC (43)								▲
2008	Scholten	*d* = −0.69 (−1.11 to −0.28)	Discrimination (meaning-prosody stroop test)	SP (48)-HC (46)								
2010	Roux	*d* = −0.45 (−1.06 to 0.16)	Discrimination (meaning-prosody stroop test)	SP (21)-HC (21)								▲
2013	Ito	*d* = −0.44 (−0.94 to 0.05)	Discrimination (meaning-prosody stroop test)	SP (28)-HC (37)								▲
2013	Iwashiro	*d* = −0.44 (−1.02 to 0.15)	Discrimination(dichotic listening of SSC pair)	SP (22)-HC (24)								●
2014	Müller	*d* = −0.03 (−0.75 to 0.69)	Discrimination (face-prosody stroop test)	SP (15)-HC (15)								●

Note. (1) Negative effect size Cohen’s d indicates worse performance in schizophrenic patients compared to healthy control; (2) Nine out of 29 studies are reported in this table. Other studies are not reported since they did not employ discrimination paradigm; (3) SSC = stimulus with semantic content; SPP = stimulus with pure prosody (without semantic content); SP = schizophrenic patients; SAVH = schizophrenia with auditory verbal hallucination; SAH = schizophrenia with auditory hallucination; SNAH = schizophrenia without auditory hallucination; SNAVH = schizophrenia without auditory verbal hallucination; HC = healthy control; ▲ = significant difference (*p* < 0.05); ● = non-significant difference; blank = not evaluated; (4) The data were collected based on the accuracy rate reported in the included studies.
